# Interleukin gene delivery for cancer gene therapy: *In vitro* and *in vivo* studies

**DOI:** 10.22038/IJBMS.2022.66890.14668

**Published:** 2023-02

**Authors:** Mohammad Amin Azimifar, Maryam Hashemi, Nahid Babaei, Zahra Salmasi, Abbas Doosti

**Affiliations:** 1 Department of Cell Molecular Biology, Bushehr Branch, Islamic Azad University, Bushehr, Iran; 2 Nanotechnology Research Center, Pharmaceutical Technology Institute, Mashhad University of Medical Sciences, Mashhad, Iran; 3 Department of Pharmaceutical Biotechnology, School of Pharmacy, Mashhad University of Medical Sciences, Mashhad, Iran; 4 Biotechnology Research Center, Shahrekord Branch, Islamic Azad University, Shahrekord, Iran

**Keywords:** Cancer, Gene delivery vector, Immunotherapy, Interleukin, Mesenchymal stem cells

## Abstract

Cytokine-mediated cancer therapy has the potential to enhance immunotherapeutic approaches and cancer elimination plans through the endowing of the immune system by providing improved anticancer immunity. Despite the encouraging pioneer studies on interleukins (ILs), the influence of ILs-originated therapeutics is still restricted by a class of potent immunoregulatory cytokines, systemic dose-limiting toxicities, ILs pleiotropy, and administration issues. During previous years, the area of transferring genes encoding immunostimulatory ILs was fundamentally widened to overcome these challenges and expedite ILs-based tumor regression. Numerous viral and non-viral delivery systems are currently available to act as crucial elements of the gene therapy toolbox. Moreover, cell-based cancer therapies are recruiting MSCs in the role of versatile gene delivery platforms to design one of the promising therapeutic approaches. These formulated gene carrier systems can provide possible alternatives to diminish dose-limiting adverse effects, promote administration, and enhance the therapeutic activity of ILs-derived treatment modalities in cancer treatment. This review provides a discussion on the advances of ILs gene delivery systems while focusing on the developing platforms in preclinical cancer immunogene therapy studies.

## Introduction

Cytokines are small soluble proteins that are secreted by different immune and non-immune cells. They function as coordinators to maintain the growth and activity of immune system cells and regulate pathophysiological processes such as cancer, inflammatory responses, and autoimmune disorders (1, 2). In the role of essential immune regulators, cytokines may be capable of providing considerable advantages for developing anti-cancer agents as mono or adjuvant therapy. They are currently experiencing a revival in conjunction with gene and engineered-cell therapies due to the low success rates of clinical studies that investigated their functionality as a single-agent therapy (3, 4).

Cytokines are categorized by diverse subgroups that include chemokines, interferons (IFNs), tumor necrosis factors (TNFs), growth factors, and ILs (1, 5). According to the gathered massive data on cytokines in recent decades, ILs constitute a substantial and significant proportion in the progression of cancer (4, 6). IL-based immunotherapy has revolutionized cancer treatments in the past few decades. ILs are identified as the most promising certified candidates for aiding the combat of the immune system against cancer due to their crucial role in modifying the immune cells’ activities and extended number of cellular sources, cell surface receptors, and signaling networks (1, 3).

Immunogene therapy approaches implicate the ensuing of both localized and systemic immune responses for attacking cancer cells (7). In comparison with short half-life therapeutic protein therapy, several studies highlighted the enhancement of host immune responses by the gene transfer of ILs against developing tumors (8). Gene transfer of ILs that encode therapeutic proteins can stand as a superior tool over the conventional recombinant protein therapy due to its capability to induce a continuous expression of more “natural” protein levels within the tumor environment to stimulate or suppress the activities of certain immune cells without causing any severe dose-limiting, and potentially life-threatening systemic, toxicity (8, 9). Furthermore, the constant high levels of ILs that occur after the introduction of an IL plasmid may result in the inducement of robust paracrine effects and improve macrophage/T-cell infiltration and other immunologic mediators within the tumor site to facilitate tumor eradication, which is impracticable with single- or multiple-bolus doses of the recombinant protein (9, 10).

A principal feature of multiple gene therapy approaches for cancer treatments is the application of gene delivery “vectors” for the local and efficient delivery of therapeutic genes. These delivery vehicles are grouped as viral and non-viral carriers to deliver genes that are usually transferred to the tumor site(s) through a local administration. Many different gene therapy carriers have been long used for the transfer of ILs genes (11). Interestingly, mesenchymal stem cells (MSCs) were extensively exploited for gene delivery purposes, particularly ILs genes. A large number of investigations on ILs-based immunogene therapy are indicative of its potential in several preclinical and also clinical experiments (11, 12). This review aimed to highlight various ILs-based gene delivery approaches while describing the current state of viral and non-viral strategies and MSCs toward the improvement of ILs-based cancer therapy. 

***Interleukins in cancer therapy***

In principle, tumor cells induce minimum immune responses in the tumor microenvironment due to their lack of expression of costimulatory molecules along with class I and II MHC molecules, which disables the triggering of tumor antigen-specific T-cell responses (6, 13). Antitumor ILs-mediated signaling at the tumor site can result in the direct enhancement of tumor cell recognition, while stimulating tumor antigen presentation, T lymphocytes and natural killer (NK) cell survival, infiltration, and hyperactivation, in order to destroy the tumor cell through stimulation of a potent tumor-directed immune response (14, 15). Several ILs are capable of promoting the activation, proliferation, and differentiation of tumor-specific CD4+ T cells into the Th1 cells that can secrete ILs and boost a host antitumor immune response (6, 14). The therapeutic potential of ILs was exploited for cancer therapy in the past few decades due to their particular immunosurveillance regulatory properties and possession of a vast signaling network for cancer development, progression, and control (4, 13). Numerous preclinical models were conducted on the broad antitumor activity of ILs, which led to a number of cytokine-based cancer therapy clinical trials that mainly implicated patients with advanced cancers (14, 15).

ILs are engineered and modified to enhance the half-life of drugs via progressive release of the active drug from a conjugated format (polymers or Fc tags), increase the toxicity of a toxic protein through receptor targeting being express locally at the tumor sites, elevating targeted therapy and reducing side effects, and capability of being delivered in a targeted manner by coupling of interleukins to tumor-targeting antibodies. On the other hand, adoptive cell therapy (ACT) approaches including T cells redirected for antigen-unrestricted cytokine-initiated killing (TRUCKs), chimeric antigen receptor (CAR), dendritic cell (DC) vaccine adjuvant, and ACT adjuvant are among the promising strategies being used to increase the translation of ILs therapeutics. (reviewed in (4)). 

Next to the continuously increasing rate of basic/translational research on the therapeutic potential of ILs (4, 15), statistical findings indicated that a large number of clinical treatments by ILs for several human cancers ended up with major challenges mainly in terms of effectiveness and toxicity, and required further cytokine modifications (1, 16). These problems were partly overcome by the design of gene delivery systems and the advancements of nanomaterials in animal experiments, which provided feasible routes for the selective transferring of therapeutic ILs to target locations with minimized systemic adverse effects (6, 16).

***Viral and non-viral gene delivery vectors***

**Figure 1:**
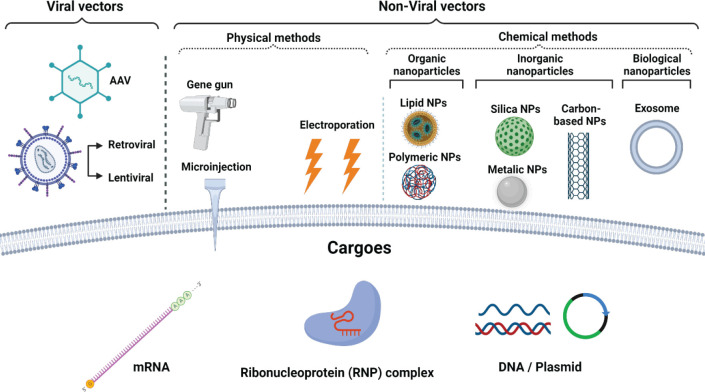
Schematic of diverse gene delivery vectors based on viral and non-viral methods. Created by BioRender.com

The vast variety of employed gene transfer methods is broadly classified as viral and non-viral carriers (Figure 1) (17). Viral gene delivery platforms consist of replication-deficient viruses for performing the transfer of desired genes to the target cells. Adenoviral, retroviral, and lentiviral carriers are commonly used for virus-based gene delivery methods (18). There are certain advantages offered by the viral platforms when compared with non-viral methods, which include the facilitation of host cell transduction with remarkable efficiency and permanent expression of delivered genes. However, the main drawbacks of using viral-based gene delivery systems are immunogenicity and toxicity concerns, insertional mutagenesis (genome integration), and the difficulty to optimize its industry-scale production (19, 20).

Non-viral vectors are divided into physical and chemical methods that include microinjection, electroporation, gene gun, cationic polymers, lipids, inorganic particles, exosomes, and different types of conjugated nanoparticle-originated complexes (18, 21). Non-viral gene delivery strategies were formulated as an alternative to viral vectors due to their higher safety and superior benefits over the viral platforms, such as low cytotoxicity, low immunogenicity, and low mutagenesis, as well as simple and low-cost large-scale production (19, 22). These advantages elevated the number of performed studies on gene therapy for conducting clinical trials through application of non-viral gene delivery systems. However, the translation feasibility of non-viral gene transfer methods was partially successful, while some critical challenges were induced by their poor transfer efficiency and specificity, which thereby lowered the duration of gene expression in their transgenes (19, 21). As displayed in Table 1, various gene delivery carriers based on viral and non-viral methods were designed for the transfer of ILs genes that majorly implicate chemicals-derived non-viral strategies. The following sections provide further detailed discussions on these areas.

***MSCs as versatile gene delivery carriers for immunogene therapy***

**Figure 2:**
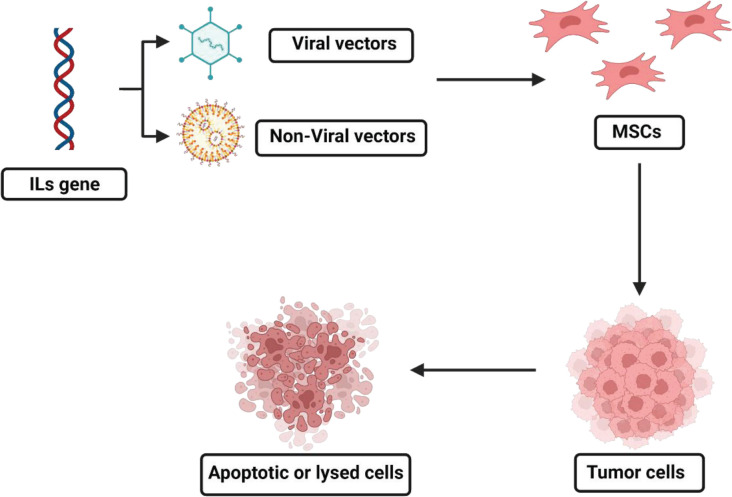
MSCs as vectors for delivery of ILs genes. Created with BioRender.com

Considering the intrinsic attributes of stem cells with their tremendous therapeutic potential, an extended rate of interest is being invested in the application of MSCs as some of the most advantageous multipotent stromal cells in cell-based gene therapies (Figure 2) (44, 45). It is feasible to significantly expand the range of disorders, for which MSCs may be useful as a therapeutic tool, through employment of rationally engineered MSCs to either boost their intrinsic synthesis of particular targeted peptides/proteins or to trigger their production of many other new proteins beyond their original common range (44, 46, 47).

The chances of exerting cell-based gene delivery systems for achieving long-term therapeutic outcomes were heightened through availability of genetically modified MSCs that are designed for gene transfer (45, 48). As remarkable delivery carriers of genes, MSCs proved to be ideally adaptable in both preclinical research and clinical gene therapy trials due to being self-renewable, readily transduced by the majority of viral vectors, and easily expandable ex vivo, while having the ability to specifically migrate to tumors, lower immunogenicity, and finely engraft within multiple tissues (44-46, 49).

Presently, MSCs were employed as carriers for targeted delivery and local production of different growth and transcription factors, suicide genes, and cytokines with various potential clinical platforms (48, 50). In this regard, several cancer models were studied to better comprehend the usage of MSCs as promising delivery tools, which were engineered to express suicide gene thymidine kinase (TK), IFNa, IFNb, IFN-γ, tumor necrosis factor-related apoptosis-inducing ligand (TRAIL), chemokine CX3CL1, thrombospondin 1(TSP-1), and other anti-cancer agents (46, 51). Subsequent to administration of transfected MSCs to tumor-bearing animals, the desired anti-cancer agents were locally produced at tumor sites to promote the annihilation of cancer cells, which resulted in inhibiting tumor cell growth and enhancing the chances of survival (48, 52).

Accordingly, the application of MSCs for transferring ILs genes proved to be more effective in ILs delivery to tumor tissues and stands as a sensible and prospective strategy for the cellular-based immunotherapy of different types of tumor (Table 2) (49). Various studies on the employment of MSCs for ILs gene delivery are provided below.

***Interleukin-2***

Considering its role in activating the immune system of cancer patients, application of interleukin-2 (IL-2) was approved for treatment of patients with metastatic renal cell carcinoma and malignant melanoma (3, 75-77). The antitumor effect of IL-2 immunotherapy is probably derived from its ability to promote proliferation, differentiation, and survival of immune cells that include activated T, NK, and B cells in vivo, which consequently induces tumor regression and inhibits tumor growth (6, 78, 79). In addition to its exertion in melanoma and renal cell carcinoma, the potential of IL-2 for performing cancer immunotherapy in other solid tumors, such as colorectal and non-small cell lung cancer (NSCLC), was investigated and achieved encouraging outcomes (3, 76). 

Viral vectors are extensively applied for the cytokine-based gene therapy of different cancers. Preclinical studies implicated the insertion of the IL-2 gene into retroviral and adenoviral vectors, and also, a pioneer study investigated the application of a retroviral vector for the delivery of IL-2 gene vector in order to cure a fibrosarcoma animal model. Authors reported that the gene transfer-mediated localized production of IL-2 can generate some remarkable in vivo antitumor immune responses (24). In the study by Slos et al., mastocytoma mice models were intratumorally administered with IL-2 encoding adenoviral vector, which resulted in increasing the levels of CD8+ cells and NK cells which consequently promotes tumor regression and survival (23).

There are some studies that indicated the efficiency of MSCs in harboring the IL-2 gene as a promising approach for the treatment of malignancies. For this purpose, bone marrow-derived MSCs were transduced with adenoviral and retroviral vectors to test their delivery potency in glioma and melanoma animal models, respectively. Their outcomes approved the effectiveness of MSCs in being exploited for transferring IL-2 and activating antitumor immune responses (53, 54). In another work, amniotic fluid MSCs were employed to investigate targeted ovarian cancer therapy, which required the usage of Lipofectamine 2000 to transfect MSCs with a plasmid that contained fluorescent IL-2. Then, nude mice with ovarian cancer were intravenously injected with IL-2 gene-expressing MSCs. The outcomes were indicative of the successful functionality of derived MSCs from the amniotic fluid as a delivery carrier of IL-2 to the desired tumor sites (56).

***Interleukin-4***

Interleukin-4 (IL-4) is a tumor immunology regulator cytokine that exhibited strong anti-tumor immunity in preclinical experiments, such as the case of Kaposi sarcoma cells (16, 80). In addition, it can act as an attractive tumor therapy agent to reinforce the efficacy of cancer immunotherapy due to its potential anti-tumor effects (3, 81). Okada et al. reported the transduction of Rat 9L gliosarcoma cells by the usage of retroviral vectors containing the IL-4 gene for performing the delivery and efficient local production of IL-4. After the genetic modification of 9L gliosarcoma cells to express IL-4, syngeneic rats received the intracranial tumor injection of the-4 producing cancer cells, which almost resulted in the death of all the rats by tumor growth. In another attempt, authors immunized rats with the intradermal injection of IL-4-producing Rat 9L gliosarcoma cells. They reported that as a result of prophylactic immunization, the IL-4-transfected tumors inhibited angiogenesis and enhanced the survival of rat models, which is a sign of therapeutic immunity to the established gliomas in the central nervous system (25).

***Interleukin-10***

As a homodimeric 17–20 kDa glycoprotein, Interleukin-10 (IL-10) is predominantly produced by innate and adaptive immune cells (15, 77). The extending evidence confirmed the stance of IL-10 as a pleiotropic immunoregulatory cytokine that is known for its potent anti-inflammatory and cytotoxic T lymphocyte-stimulating functions, which fosters the assumption of its ability in performing anticancer activity mainly through the CD8+ T cell-mediated antitumor immunity (4, 82, 83). 

Bone marrow-derived MSCs were utilized for the targeted tumor delivery of the IL-10 gene to evaluate the anticancer potential of IL-10. Related research attempted to transduce MSCs with lentivirus expression vectors to harbor IFN-γ and IL-10. Apparently, the co-expression of IFN-γ and IL-10 caused the suppression of hepatocellular carcinoma tumors in male Sprague-Dawley rats. According to the gathered data, this effect was associated with the alteration of the MAPK pathway that occurred by the activation of p38 and JNK, and the inactivation of ERK (57). In another strategy, Lipofectamine was applied to transfect MScs with a plasmid containing the IL-10 gene for performing the IL-based gene therapy of pancreatic cancer. Their results indicated that the transferred IL-10 via MSCs was able to prevent the inducement of angiogenesis and tumor growth while increasing the survival of tumor-bearing animals. There might be a possible correlation between these findings and the indirect suppressed production of pro-inflammatory cytokines IL6 and TNF-α (58).

***Interleukin-12***

In the form of a pro-inflammatory and immunostimulatory cytokine, Interleukin-12 (IL-12) is a heterodimeric protein consisting of two subunits (P35 and P40 which represent the approximate molecular weight) that are linked by a disulfide bond (84). IL-12 is predominantly released by antigen-supplying cells, natural killer cells, and lymphocytes to progress through antigen stimulation and induce a wide range of essential events for immune responses. In comparison with different cytokines, IL-12 is a potent candidate for cancer immunotherapy due to its significant roles in the reduction of tumor progression which include supporting tumor-associated macrophages, exhibiting superior anti-angiogenic activities, and triggering anti-inflammatory responses (7, 85). 

The systemic administration of transferred IL-12 as a recombinant protein was limited by its instability, potential systemic toxicity, and lower efficacy; therefore, it was necessary to establish various forms of gene therapy carriers to increase the local concentration of this cytokine in the tumor microenvironment. This method can maintain the expression of IL-12 at low levels and eventually reach the baseline, which provided outstanding therapeutic benefits for clinically relevant animal models (86, 87). However, several preclinical studies on the diverse delivery systems of IL-12, from viral and non-viral transfer to MSCs-based strategies, exhibited the stance of IL-12 as one of the robust anticancer cytokines (84, 87).

Up to this date, a wide variety of viral and nanoparticle-based delivery systems were designed for the in vivo delivery of IL-12 genes to targeted tumors. In this regard, Rodríguez et al. examined the antitumor activity of 4-methylumbelliferone(4Mu), in the role of a hyaluronan synthesizing inhibitor, in combination with an adenovirus vector that encoded the IL-12 gene. According to the results, this approach can cause serious anti-tumor effects and significantly increase the survival rate of hepatocellular carcinoma animal models (26). In another work, adenovirus-mediated IL-12 genes were transferred to explore the effects of combined adenovirus-mediated suicide gene and IL-12 gene therapy in an animal prostate cancer model, which exhibited the optimistic results of this strategy for the treatment of cancer (27).

In an in vitro and pilot study, Khalvati et al. reported the design of a succinic anhydride-conjugated polyethylenimine (PEI) delivery platform for IL-12 gene therapy and claimed the promising functionality of this engineered PEI vector for ILs gene delivery (31). Moreover, polymetformin-derived nanoplatform micelleplexes were applied as a combined strategy to co-transfer doxorubicin (DOX) and IL-12 plasmid DNA for the management of metastatic breast cancer. The data of this chemo-gene combination therapy in the mice model of 4T1 breast cancer lung metastasis was indicative of an enhanced antitumor and anti-metastatic efficacy (29). In another interesting study, PQDEA was used as a polymer to efficiently transfect both cancer cells and tumor-associated macrophages with IL-12 genes. According to their results, this intravenous injected non-viral vector forced the tumor to produce high amounts of IL-12 and triggered anticancer immune responses with minimal cytotoxicity. Their study was claimed to be the very first assessment with the simultaneous exertion of tumor cells and tumor-associated macrophages as a delivery system (28). 

In regards to the consideration of MSCs as versatile delivery machines, Ryu et al. reported the usage of umbilical cord blood-derived MSCs (UCB-MSCs) as a carrier to transfer therapeutic IL-12 genes toward the targeted glioma. They transduced UCB-MSCs with a tetrameric cell-permeable peptide (4HP4)-originated adenoviral vector. As a result, the intratumoral injection of UCB-MSC-IL-12 caused a noticeable increase in the survival rate of mice and decreased the growth of tumors when compared with the control group. They also discovered that a local increase in the IL-12 levels leads to T-cell infiltration and secretion of interferon-γ in intracranial gliomas (65). The work of Duan et al. implicated the transferring of IL-12 genes by the bone marrow-derived MSCs in Ewing’s sarcoma mice model. Bone marrow-derived MSCs were infected with an adenoviral vector that harbored IL-12 genes and subsequent to the intravenous injection of engineered MSCs, the local expression of IL-12 was confirmed by observing the inhibition of tumor growth (47).

Seo et al. reported the observance of remarkable antitumor immune effect and potent tumor antigen-specific T-cell activity from the intratumoral delivery of MSCs producing IL-12. In comparison with IL-12-expressing adenoviruses, there was higher stability in the localized generation of IL-12 by MSCs in the tumors of mice with metastatic and solid tumors. The findings indicated the superior effectiveness of MSCs as ILs gene delivery vehicles compared with adenoviruses, as well as approving the approach of intratumoral administration as the best way of inducing potent tumor-specific T-cell responses to achieve anti-metastatic effects and inhibiting the growth of solid tumors (60). Another study investigated the impacts of transduced MSCs via lentivirus-expressing IL-12 vector on the malignant ascites of mice samples, which resulted in the high production of IL-12 in the ascites of IL-12 MSC-treated mouse and exhibited a potent chemotactic effect on dendritic cells. In addition, IL-12 MSCs prolonged the duration of survival and increased its rate in the mice models by reducing the count of red blood cells and the volume of ascites. The application of IL-12 MSCs in mice with malignant ascites was also observed to be nontoxic without inducing any side effects (59).

In another research, Kułach et al. presented a delivery system based on IL-12-producing MSCs to enhance the antitumor immune responses against melanoma-bearing mice models. According to their results, the intravenous administration of MSCs-secreting IL-12 led to the occurrence of remarkable tumor inhibition and diminished the number of metastasis in mice without causing any toxicity. Authors claimed that the antitumor benefits can be related to the pleiotropic features of the released IL-12 by engineered MSCs (62). In a recent investigation by the author of the current study, a poly-(amidoamine) (PAMAM) (G5) was exploited to transfect adipose tissue-derived MSCs by using a plasmid encoding IL-12 gene. The outcomes exhibited the low toxicity of alkyl-peptide PAMAM, which reveals its superior potential for transfecting MSCs with IL-12 gene compared with PAMAM-peptide, PAMAM-alkyl, and PMAMAM. The gathered data also indicated the greater potency of engineered MSCs to migrate into cancer cells when compared with normal MSCs. Therefore, it is expected that the efficient transfection of engineered MSCs with alkyl-peptide PAMAM and the implication of the IL-12 gene as a carrier would aid the activation of immune responses in animal and clinical studies (66).

More recently, Dehshahri and colleagues developed a cationic polysaccharide-based IL-12 delivery system, labeled as N-[(2-hydroxy-3-trimethylammonium)propyl] chitosan salt (HTCS) and evaluated the transfection efficiency and cytotoxicity of prepared polyplexes. The obtained outcomes confirmed the sufficiency of the generated platform as a potential IL-12 gene carrier (40).

***Interleukin-15***

Interleukin-15 (IL-15) is mainly expressed by activated myeloid cells that are structurally and functionally similar to IL-2. It is capable of promoting more memory maintenance in the CD8+ T cells, which may be crucial for persisting long-term anti-cancer immunity (3, 6, 88). A number of preclinical cancer experiments performed extensive investigations on the potential of IL-15 as an antitumor agent that is mediated by NK cells and T lymphocytes, which recently entered trial studies for the mediation of cancer regression (15, 77, 89). However, recent investigation indicated the benefit of targeted gene transfer approaches in providing a higher local dosage of IL-15 in order to result in more effective cancer immunotherapy. 

In regards to pancreatic cancer, it can be stated that the conduction of gene therapy by the usage of umbilical cord blood–isolated MSCs, transduced with the lentiviral vector that harbors IL-15 genes, can hinder tumor progression and prolong the survival time of tumor-bearing mice following systemic administration. Accordingly, the expression of IL-15 was significantly up-regulated in pancreatic xenograft tumor tissues, confirming the tumor-specific migration ability of MSCs-IL-15. In conformity to further analysis, the antitumor activity of MSCs-IL-15 originated from the functionality of NK and CD8+ T cells (67). 

***Interleukin-18***

In the form of a 24 kDa polypeptide, Interleukin-18 (IL-18) induces IFN-γ production by the involvement of NK and CD8+ T cells and enhances their cytolytic activities. Moreover, this cytokine is structurally and functionally related to the IL-1 cytokine superfamily (6, 15). IL-18 is involved in many biological activities such as the process of immune responses by T-helpers type 1 and type 2 and inducement of innate immunity (90). Moreover, the emerging insights into the antitumor immunity effects of IL-18 were facilitated by the reduction of tumorigenesis, macrophage activation, inhibition of tumor angiogenesis, and induction of tumor cell apoptosis (88, 91). Considering the results of related studies, glioma, breast, and bladder carcinoma prognosis are responsible for increasing the levels of IL-18 in circulation (70). Their data indicated that the level of IL-18 in patients with breast and liver cancer that also bear metastases is significantly increased in comparison with the serum levels of normal individuals (92). These biological performances signify that IL18 may be beneficial for cancer immunogene therapy, which was also approved by their substantial anticancer functionality in preclinical animal experiments (4, 6). 

In another study, different cationic emulsions were exerted as non-viral delivery systems for the transferring of IL-18 genes to the lung cancer cells. The authors compared the efficiency of this developed carrier with Lipofectamine and reported the superiority of this cationic emulsion-derived carrier in terms of IL-18 gene distribution in the liver and lung, approving its promising stance as an IL gene delivery system (10). The adenoviral-mediated transduction of MSCs with the IL-18 gene was attempted in the work by Xu *et al*., which reported the effects of intratumoral injection of MSCs-secreting IL-18 to glioma-bearing rats in significantly suppressing the tumor growth and promoting the rate of survival (70). 

The in vitro experiment of Liu *et al*. reported the utilization of lentiviral vectors for transferring IL-18 genes to human MSCs derived from the umbilical cord in order to investigate the effect of MSCs-secreting IL-18 on the in vitro migration, invasion, and growth of HCC1937 and MCF-7 breast cancer cells. MSCs-secreting IL-18 was observed to considerably restrict the invasion, proliferation, and migration of HCC1937 and MCF-7 cells (68). In another assessment, the same group designed an in vivo platform for their primary study by using mice with breast cancer and evaluated the factors of tumor size, proliferation, and metastasis. The results displayed the occurrence of a decrease in the metastases and proliferation of the MSCs-secreting IL-18 group when compared with the control groups. Apparently, the MSCs-secreting IL-18 cells were able to restrain tumor cell proliferation and enhance the antitumor efficacy by triggering immune cytokines and immunocytes (69).

***Interleukin-21***

Interleukin-21 (IL-21) is mainly expressed by CD4+ T and NKT cells and is recognized as a potent regulatory cytokine of common γ IL-2, IL-4, and IL-15 family, which is responsible for managing the innate and adaptive immune systems (88, 89). IL-21 can destroy cancerous and virally infected cells by affecting NK and cytotoxic T cells (93, 94). Considering its capacity to boost the cytotoxic activity of CD8+ T cells and NK cells, IL-21 demonstrated therapeutic activity in preclinical animal models and recently progressed to phase 2 of clinical trials for the treatment of cancer (15, 41, 77, 89).

Kim-Schulze and co-workers applied Lipofectamine 2000 for the introduction of IL-21 genes into the melanoma cells for the purpose of providing the sustained and local production of IL-21. According to their discoveries, the intratumoral microenvironment expression of IL-21 increased the level of CD8+ T–cell-related responses and led to the suppression of tumor growth. They also observed a signiﬁcant increase in the local proliferation of CD8+ T cells along with a decrease in CD4+ regulatory T cells *in vivo* through the non-viral delivery of IL-21 (41).

Concerning the role of MSCs as gene delivery carriers, Hu et al. tried the application of umbilical blood mononuclear cell-isolated MSCs for the delivery of IL-21 genes to the epithelial ovarian cancer animal models to investigate animal survival and tumor volume subsequent to the intravenous injection of MSCs-producing IL-21. The results indicated the considerable elevation of interferon-γ secretion and cytotoxicity of NK cells in the treated mice when compared with the control animals group while observing the great reduction of tumor growth and increased animal survival as a result of transferring IL-21 (95).

***Interleukin-24***

As a member of the IL-10 cytokine gene family, Interleukin-24 (IL-24) is an immunoregulatory cytokine produced by activated monocytes and T cells (6, 72). The overexpression of IL-24 can suppress the growth of a broad spectrum of tumor cells by causing apoptosis through the regulation of intracellular and extracellular signaling mediators (72, 74). Several researches approved the stance of IL-24 as a novel and effective candidate for tumor immunotherapy due to its abilities in mediating direct anticancer activities and also impeding tumor angiogenesis and metastasis (73, 96). Considering its immunostimulatory properties and the outcomes obtained from both pre-clinical and clinical studies, the emergence of IL-24 as a potent tumor-inhibiting agent is undeniable, however, the clinical usage of this agent is practically limited by the deficiencies of targeted and efficient delivery systems (6, 72). To provide a direct path towards a more effective translation of IL-24 therapy from laboratory trials to clinical application, it is of fundamental importance to either increase the overall administration of local IL-24 or facilitate its specific delivery to the tumor’s location (72).

In one study, human umbilical cord-originated MSCs were used to transduce with an adenoviral vector for transferring IL-24 to lung cancer cells and tumors. The transfer of this cytokine to A549 lung cancer cells resulted in inhibiting the cell cycle and induction of apoptosis. In addition, they reported the effects of IL-24 on the signaling pathways of ERK-1/2, AKT, and JNK, as well as its ability to inhibit both tumor angiogenesis *in vitro *and* in vivo* and the tumor growth of tumor-bearing mice. Collectively, their outcomes indicated the effectiveness of targeted IL-24 transferring through MSCs for the management of cancer (72).

In another attempt to employ MSCs for the targeted delivery of the IL gene, umbilical cord-originated MSCs were ex vivo transduced with lentiviral vectors to perform the IL-24 gene therapy of glioma. Their data demonstrated the migratory capability of MSCs-secreting IL-24 in both *in vitro *and *in vivo* situations. The results of the animal study showed the inhibition of tumor growth caused by administration of MSCs-secreting IL-24 (73). More importantly, Wu *et al*. exploited the induction of pluripotent stem cells (iPSCs)-derived MSCs for performing the targeted delivery of the IL-24 gene to melanoma tumor animal model. The iPSCs-derived MSCs were transduced with a non-viral vector, minipHrn, to evaluate their tumor migratory and antitumor functionality. The outcomes exhibited tumor suppressive activity and confirmed the tumor migration of iPSCs-derived MSCs-producing IL-24. Moreover, the injection of iPSCs-derived MSCs-producing IL-24 resulted in elevating the expression of Bax and cleaved caspase-3, while causing the down-regulation of Bcl-2, in the animal experiments (74).

***Interleukin-27***

As a member of the IL-12 cytokine family, Interleukin-27 (IL-27) is a pleiotropic two-chain cytokine that is primarily expressed by antigen-presenting cells (APCs) including dendritic cells (DCs) and macrophages (97-100). There is much evidence from preclinical cancer models that indicate its potential in exhibiting antitumor immunity against highly immunogenic tumors through a variety of mechanisms, which is due to its efficient contribution to the antigen-presenting processes, regulating T helper cell differentiation, and decreasing the rate of angiogenesis and metastasis (6, 101).

Adeno-associated viruses were harnessed as gene delivery vehicles for conducting the IL-27-immunogene therapy of melanoma, colon cancer, breast cancer, and plasmacytoma. Viral carriers that contained IL-27 genes were intramuscularly injected into 2 sites of mice’s hind legs. According to their findings, treatment with Adeno-associated virus-expressing IL-27 led to the depletion of Tregs in the tumor microenvironment. The outcomes of the plasmacytoma animal model showed the involvement of IL-10 throughout the tumor suppression of Adeno-associated virus-IL-27 (42). In another assessment on immunogene therapy, a fusion of IL-27-secreted luciferase was constructed for investigating its targeted anticancer gene therapy activity as a novel cytokine-based biological (Nluc-27) platform. This in vitro prostate cancer model was capable of decreasing cancer cell proliferation by implication of targeted Nluc-27. The authors claimed the promising stance of this strategy as a delivery design of second-generation IL-27 for performing the targeted gene therapy of other targeting domains and cancer cells (43).

***Challenges and future perspective***

Cytokine-based cancer immunotherapy attracted the interest of many among the several available cancer prevention and treatment modalities. The present review provided a list of various available viral and non-viral delivery platforms for ILs gene transfer to cancer cells and animal tumor models. In this context, different virus-originated vectors and polymer-based nanoparticle carriers were fabricated with unique properties for being applied in the local delivery of genetic materials to the target cells. More interestingly, the extensive utilization of MSCs as vehicles is considered for performing an efficient gene delivery to specific cells or tissues due to their higher effectiveness and lack of undesirable consequences and systemic toxicity, which would be more suitable to be classified as a versatile category of non-viral vectors. Herein, we focused on preclinical studies as the primary designs of this field to compare the basic concepts of the developed transferring strategies.

Despite some advantages, the introduced gene delivery constructs are conflicted with serious limitations that prevent their applicability in clinically relevant purposes. For instance, their systemic administration may be challenged by cytokine pleiotropy, poor pharmacokinetics, rapid degradation, harmful side effects, and dose-limiting toxicities. In addition, the immunocompetent mouse, which is the common preclinical animal model, has some fundamental differences with human cases in immunogenicity, rate of tumor growth, cellular combination, receptor expression, cytokine responses, vascularization, and immune infiltration. Additional factors that might reduce the efficacy of cytokine gene therapy include the prevented activity of effector immune cells due to the presence of immunosuppressive factors at the tumor site, as well as the lack of the immune system’s capacity to recognize tumor antigens.

On the other hand, the solo application of immunotherapy cannot provide the required efficiency in most cases for destroying solid tumors, which is due to the down-regulation of host immune responses and their escape from immune surveillance. Therefore, the complete annihilation of a high-grade solid tumor is quite difficult for cytokine delivery systems. 

These phenomena can affect the efficacy of platforms. The methods with the synergistic effect can address this issue. One of the suggested approaches is to use the combination of radiation therapy and cytokine-based immunotherapy. Radiation therapy exhibited a strong potency in local tumor therapy through eradication of tumors’ defensive manners, induction of immune-stimulatory gene expression, facilitating the extravasation and activation of immuno-effector cells, and triggering the process of apoptosis. Moreover, this method demonstrated a synergistic efficacy between chemotherapy and cytokine-based immunotherapy at lower doses. Thereby, IL-based cancer therapy may be able to improve the potential of other therapeutics by induction of antitumor immunity, which would provide certain benefits for therapy-resistant patients. Finally, it is essential to further assess the exertion of smart multifunctional carriers that contain chemotherapeutic drugs and genes in order to achieve better therapeutic effects.

Other strategies for clinical development of ILs delivery include maintaining the efficacy of platforms by minimizing the exposure of plasma, reducing the toxicities, assuring the stability of formulation in storage, and simplifying their administration. In this line, it is absolutely beneficial to attempt to better understand immuno-pharmacology, modulate the pharmacokinetics and pharmacodynamics of delivery systems without changing their biodistribution, and fully understand their mechanisms to modulate the therapeutic effect and toxicity. Therefore, the next steps of research must further investigate the optimization of more effective modalities and delivery strategies for achieving a clinically feasible and safe gene-transferring method with satisfying therapeutic outcomes.

**Table 1:** Viral and non-viral ILs gene delivery systems

**Interleukin gene**	**Delivery platform**		**Cancer type**	**Study type**	**References**
IL-2	**Viral**	Adenovirus vector	Mastocytoma	*In vivo*	(21)
		Retrovirus vector	Fibrosarcoma	*In vitro*/*In vivo*	(22)
IL-4	**Viral**	Retrovirus vector	Gliosarcoma	*In vitro*/*In vivo*	(23)
IL-12	**Viral**	Adenovirus vector	Hepatocellular carcinoma	*In vitro*/*In vivo*	(24)
			Renal cortical adenocarcinoma	*In vivo*	(15)
			Prostate cancer	*In vivo*	(25)
	**Non-viral**	Poly (N-[2-(acryloyloxy)ethyl] -N-[p-acetyloxyphenyl]- N,N-diethylammoniumchloride) (PQDEA)	Pancreatic ductal adenocarcinoma, melanoma, mammary carcinoma	*In vivo*	(26)
		Hyaluronic acid-polymetformin micelleplexes	Breast cancer	*In vivo*	(27)
		Diethylene triamine penta-acetic acid (DTPA)-conjugated PEI	Hepatoma	*In vitro*	(28)
		Succinic anhydride-conjugated PEI	Hepatoma	*In vitro*	(29)
		Polymeric PEI-polyethyleneglycol- cholesteryl chloroformate	Ovarian cancer	*In vivo*	(12)
		Chitosan	Fibrosarcoma	*In vivo*	(30)
		Lipofectamine 2000	Fibrosarcoma	*In vitro*/*In vivo*	(31)
		Poly [D,L-2,4-diaminobutyric acid] (PDBA)	Melanoma	*In vivo*	(32)
		*Cationic Sendai* virus fusion protein and hemagglutinin-neuraminidase protein virosomes	Ovarian adenocarcinoma	*In vitro*/*In vivo*	(33)
		Folate-modified liposome	Colon cancer	*In vivo*	(34)
		Poly[α-(4-aminobutyl)-L-glycolic acid] (PAGA)	Colon adenocarcinoma	*In vivo*	(35)
		DOTAP modified mPEG– poly(ε-caprolactone) cationic micelles	Colon cancer, lung cancer	*In vivo*	(11)
		Acrylates-modified polyamidoamine (PAMAM) dendrimers	Hepatoma	*In vitro*	(36)
		Poly[α-(4-aminobutyl)-L-glycolic acid] (PAGA)	Colon adenocarcinoma	*In vitro*/*In vivo*	(37)
		N-[(2-hydroxy-3-trimethylammonium)propyl] chitosan salt (HTCS)	Breast cancer, hepatoma	*In vitro*	**(** 38 **)**
IL-18	**Non-viral**	Cationic emulsions	Lung cancer	*In vitro*/*In vivo*	(13)
IL-21	**Non-viral**	Lipofectamine 2000	Melanoma	*In vitro*/*In vivo*	(39)
IL-27	**Viral**	Adeno-associated virus vector	Melanoma, colon cancer, breast cancer, plasmacytoma	*In vitro*/*In vivo*	(40)
	**Non-viral**	Lipofectamine 2000	Prostate cancer	*In vitro*	(41)

**Table 2:** MSCs as ILs gene delivery platforms

**Interleukin gene**	**Carrier for IL gene delivery to stem cells **		**Stem cell type**	**Cancer type**	**Study type**	**References**
IL-2	**Viral**	Adenovirus vector	Bone marrow-derived MSCs	Glioma	*In vivo*	(51)
		Retrovirus vector		Melanoma	*In vivo*	(52)
		Lentivirus vector	Human adipose tissue-derived MSCs	Prostate cancer, neuroblastoma, lung adenocarcinoma	*In vitro*	(53)
	**Non-viral**	Lipofectamine 2000	Amniotic fluid-derived MSCs	Ovarian cancer	*In vivo*	(54)
IL-10	**Viral**	Lentivirus vector	Bone marrow-derived MSCs	Hepatocellular carcinoma	*In vivo*	(55)
	**Non-viral**	Lipofectamine 2000		Pancreatic cancer	*In vivo*	(56)
IL-12	**Viral**	Lentivirus vector	Bone marrow-derived MSCs	Fibrosarcoma, hepatoma	*In vivo*	(57)
		Adenovirus vector		Melanoma, cervical cancer	*In vivo*	(58)
				Ewing's sarcoma	*In vivo*	(45)
				Glioma	*In vivo*	(59)
				Melanoma	*In vivo*	(60)
		Retrovirus vector		Glioblastoma	*In vivo*	(61)
				Melanoma	*In vivo*	(62)
		Adenovirus vector	Human umbilical cord blood-derived MSCs	Glioma	*In vitro*/*In vivo*	(63)
	**Non-viral**	Alkyl/LMWP-modified poly-amidoamine	Adipose tissue-derived MSCs	Hepatoma	*In vitro*	(64)
IL-15	**Viral**	Lentivirus vector	Umbilical cord blood-derived MSCs	Pancreatic cancer	*In vivo*	(65)
IL-18	**Viral**	Lentivirus vector	Umbilical cord blood-derived MSCs	Breast cancer	*In vitro*	(66)
				Breast cancer	*In vivo*	(67)
		Adenovirus vector	Bone marrow-derived MSCs	Glioma	*In vivo*	(68)
IL-21	**Non-viral**	Lipofectamine 2000	Human umbilical blood mononuclear cell-derived MSCs	Epithelial ovarian cancer	*In vivo*	(69)
IL-24	**Viral**	Adenovirus vector	Umbilical cord-derived MSCs	Lung cancer	*In vitro*/*In vivo*	(70)
		Lentivirus vector		Glioma	*In vitro*/*In vivo*	(71)
	**Non-viral**	Non-viral vector (minipHrn)	Induced pluripotent stem cells (iPSCs)-derived MSCs	Melanoma	*In vivo*	(72)

## Authors’ Contributions

MH and NB Conceived the study or design; MAA Analyzed data and prepared the draft manuscript; ZS Analyzed and interpreted the results; MH, AD, and NB Critically revised or edited the article; MH Provided supervision and funding acquisition; MAA, NB, ZS, AD, and MH Approved the final version to be published.

## Conflicts of Interest

There are no interests to declare.
